# Albumosuria

**Published:** 1910-09-17

**Authors:** 


### ALBUMOSURIA.

The most characteristic reaction of the urine in cases of albumosuria is the fact that when the specimen has been warmed to boiling point an abundant precipitate, suggesting albumin, appears during the process of heating, disappearing again before boiling point is reached. This reaction is even more striking than is the better-known test for albumose, namely, that with the nitric acid test a white ring appears at the line of junction to disappear on warming and reappear on cooling.

By far the most common cause of albumosuria, and almost the only one which produces what might be called an abundant albumosuria, is multiple myeloid tumours of bones. Traces of albumose may appear in the urine, in many febrile diseases, and in chronic suppuration, such as follows upon empyema or in connection with joint diseases, but the amount of albumose in these cases is very different from the large amount met with when there are malignant deposits in connection with the bone marrow, in which case the albumose may form an almost putty-like deposit.

A case in point has recently been recorded by Langdon Brown. A Jew, aged fifty, was admitted to hospital with a history that he had been ailing for about a year. He complained of pains all over and of loss of flesh. He was anæmic, looked ill and in pain, and he groaned whenever he was moved. No swelling of any bone or joint could be made out, though the ribs were very tender. The chest was emphysematous, and there were signs of bronchitis. Nothing else was made out, except that the urine was found to contain a trace of "Albumin" with hyaline casts. Measured by Esbach's method the amount of "albumin" rapidly increased from a trace to about two parts per thousand. There was also an abundance of phosphates. It was noted particularly that the abundant precipitate of albumin which had first appeared on warming disappeared on boiling, and this roused the suspicion that it was not albumin but albumose—the variety of proteose described by Bence-Jones. This was confirmed by the nitric acid test mentioned above. The patient grew steadily worse, and hard nodules could presently be felt attached to several of the ribs. These swellings corresponded to the points of maximum tenderness that had been previously observed and the skin over them was movable. Hard movable glands could be felt in the right axilla. In a few days death resulted from broncho-pneumonia.

The detection of albumosuria enables a diagnosis to be made before the tumours appear in some of these cases, and at a time when the character of the pain may be such as to suggest some far less serious ailment, such, for instance, as myalgia or rheumatism.

